# Auditory rhythm facilitates perception and action in children at risk for developmental coordination disorder

**DOI:** 10.1038/s41598-024-62322-6

**Published:** 2024-05-28

**Authors:** Chantal Carrillo, Andrew Chang, Hannah Armstrong, John Cairney, J. Devin McAuley, Laurel J. Trainor

**Affiliations:** 1 Department of Psychology, Neuroscience, and Behaviour, McMaster University, Hamilton, ON Canada; 2 Infant and Child Health (INCH) Lab, Department of Family Medicine, McMaster University, Hamilton, ON Canada; 3 School of Human Movement and Nutrition Sciences, University of Queensland, Brisbane, QLD Australia; 4 Department of Psychology, Michigan State University, Michigan, USA; 5 McMaster Institute for Music and the Mind, McMaster University, Hamilton, ON Canada; 6 Rotman Research Institute, Baycrest Hospital, Toronto, ON Canada; 7 Present Address: Department of Psychology, New York University, New York, USA

**Keywords:** Psychology, Health care

## Abstract

Developmental Coordination Disorder (DCD) is a common neurodevelopmental disorder featuring deficits in motor coordination and motor timing among children. Deficits in rhythmic tracking, including perceptually tracking and synchronizing action with auditory rhythms, have been studied in a wide range of motor disorders, providing a foundation for developing rehabilitation programs incorporating auditory rhythms. We tested whether DCD also features these auditory-motor deficits among 7–10 year-old children. In a speech recognition task with no overt motor component, modulating the speech rhythm interfered more with the performance of children at risk for DCD than typically developing (TD) children. A set of auditory-motor tapping tasks further showed that, although children at risk for DCD performed worse than TD children in general, the presence of an auditory rhythmic cue (isochronous metronome or music) facilitated the temporal consistency of tapping. Finally, accuracy in the recognition of rhythmically modulated speech and tapping consistency correlated with performance on the standardized motor assessment. Together, the results show auditory rhythmic regularity benefits auditory perception and auditory-motor coordination in children at risk for DCD. This provides a foundation for future clinical studies to develop evidence-based interventions involving auditory-motor rhythmic coordination for children with DCD.

## Introduction

Developmental Coordination Disorder (DCD) is a neurodevelopmental disorder defined as a marked impairment in the acquisition and performance of coordinated motor skills. DCD affects 5–6% of children across a wide range of fine and gross motor abilities and has a significantly negative impact on the execution of simple daily tasks^1^. The main deficits in DCD include poor motor performance, poor motor learning, reduced automatization of movement, poor anticipatory control, and decreased manual control compared to typically developing (TD) children^2^. Children with DCD are often described as “clumsy”, as they struggle with tasks such as handwriting, tying their shoes, using scissors, and riding a bike. These deficits are later associated with decreased academic achievement, lower self-esteem, anxiety, depression, childhood obesity, and a higher reluctance to partake in leisure activities^3–7^. DCD is viewed as chronic, and an estimated 75% of cases will continue into adulthood if there is no intervention^8^. Despite the high prevalence and negative outcomes, very little is known about DCD and how to effectively treat it.

A crucial component of motor performance is temporal coordination, and poor motor timing is one of the core deficits of DCD^9–11^. As children navigate the world and interact with their environment, they often need to temporally coordinate their movements with external stimuli, whether it be catching a ball, walking in synchrony with a friend, or moving along to music. Research on sensorimotor timing skills in children with DCD has increased in recent years^12,13^. In the visual-motor timing domain, children with DCD show deficits in intercepting moving objects^14^, perceiving visual temporal gaps^15^, bimanual tapping to a visual stimulus^16^, and synchronization of verbal responses to isochronous visual and auditory cues^17^.

Sensorimotor coordination is particularly intriguing and important in the auditory domain, as both the perception and production of speech and music involves auditory and motor systems. Specifically, a large body of evidence suggests that the perceptual processing of rhythmic regularity in speech and music involves neural networks spanning auditory and motor cortical regions, even in the absence of overt motor movement^18–24^. In a study using nonmotor perceptual tasks, children at risk for DCD have been shown to have worse thresholds for detecting auditory duration and rhythm deviations, but not pitch deviations, compared to typically developing children^25^. This auditory-motor connection is also closely tied to social interaction—for example, moving in synchrony with others along with musical rhythms increases cooperation, helping behaviours, and feelings of affiliation^26–28^. Thus, a deficit in auditory-motor skills could have consequences for perceptual, cognitive, and social development. However, whether the motor deficits among individuals with DCD are associated with poorer auditory-motor timing abilities remains a largely unexplored hypothesis^29^.

To the best of our knowledge, auditory-motor skills in DCD have only been sporadically reported in a few studies. Children with DCD are less accurate at tapping to an auditory metronome^30^, and “clumsy” children (who are likely to have DCD) have higher variability in sustaining their tapping tempo after removing auditory cues^31^. In a bimanual tapping coordination task, children with DCD are more variable and less accurate than typically developing (TD) children at coordinating with an auditory cue^32^. Children with both attention-deficit hyperactivity disorder (ADHD) and DCD showed deficits in paced and unpaced tapping tasks compared to typically developing children and children with ADHD alone^33^. It is unclear, however, how well children with DCD can synchronize with a variety of more complex auditory stimuli, and how auditory rhythm perception and auditory-motor synchronization skills relate to the general motor skills that define DCD, such as those measured by the Movement Assessment Battery for Children (MABC-2).

Rhythmic tracking behaviour (or entrainment), a key component of auditory-motor synchronization, may be a domain-general deficit in DCD. Rhythmic tracking requires prediction of the timing of upcoming auditory events. To accurately synchronize movements to an auditory stimulus, an individual must successfully predict upcoming events in the auditory stimulus, form expectations for the target movement^34^, and correct for errors or changes in the stimulus^35^. Rhythmic regularity in auditory stimuli can facilitate perception by proactively guiding attention to rhythmic events^36–38^, or be used to precisely coordinate movements^39,40^. Thus, if DCD features a deficit in rhythmic tracking, it should be observed on both perceptual tasks that require temporal tracking without an overt motor component as well as on auditory-motor synchronization tasks.

The present study is centered around the question of whether children at risk for DCD (rDCD) flexibly use rhythmic tracking of auditory temporal cues to improve (1) auditory perception of speech and (2) auditory-motor performance in comparison to TD children. The auditory perception of speech task employed distorted timing to compare speech recognition in rhythmically intact and rhythmically modulated sentences. This task, which did not involve any overt motor component, aimed to understand whether children with DCD use rhythm as a cue in the highly overlearned skill of speech recognition, and whether they could flexibly adjust when the rhythm of the speech was modulated. Specifically, we used a task in which children identified key words in target sentences (among distractor sentences) when the sentences were normally timed and when their timing was modulated. Adults perform worse on this task when the speech rhythms are modulated^41,42^, as rhythm provides a temporal cue for where to focus attention when identifying target words. However, the degree to which their performance suffers can be a measure of how flexible they are in adjusting to modulated rhythmic cues. Although previous research found that children with rDCD show poorer rhythm discrimination than TD children^25^, they did not investigate whether children with rDCD can utilize rhythmic temporal regularity to proactively improve their perception of an upcoming auditory signal. We expected children with rDCD to be less able to use the rhythmic cues in speech to their benefit, and thus perform overall worse at the task compared to TD children. We also expected children with rDCD to be less adaptable to rhythmic modulation, and for modulated speech rhythms to have a stronger negative impact on performance compared to in TD children, consistent with previous findings showing less flexibility in DCD on visual-motor timing^43^.

The auditory-motor synchronization tasks investigated whether the presence of auditory rhythmic cues would improve rhythmic motor tapping performance in children with rDCD in comparison to TD children. We examined performance on (1) tapping in synchrony with a simple auditory metronome, (2) continuation tapping (maintaining tapping behavior after the auditory rhythm stops), and (3) tapping to the beat of musical excerpts where the beat needs to be extracted from the complex rhythmic structure. We expected that children with DCD would be more variable or imprecise in their tapping compared to TD children, but that both TD and DCD groups would tap more accurately and with less variability when an auditory cue was present compared to when there was no auditory cue.

## Methods

### Participants

46 children aged 7 to 10 were recruited through collaboration with the Infant and Child Health (INCH) lab^44^. All participants were part of a longitudinal coordination and activity tracking in children (CATCH) study, and written consent was received from parents/guardians to share their MABC-2 scores. The CATCH study recruited a large number of children (n = 588) from communities in southern Ontario, and measured them every year for six years on a variety of motor tasks, including the MABC-2, which is the most widely used assessment for DCD based on an established threshold^45,46^. We identified the participants at risk for DCD following the diagnostic and statistical manual of mental disorders (DSM-5) criteria and the 2011 European Academy of Childhood Disability Guidelines for identification of children with DCD^45^. These included significant motor deficits, as assessed by the MABC-2 (participants with scores in the lower 16^th^ percentile were considered at risk for DCD) and that these motor deficits were not due to existing intellectual disability (all participants had IQ scores above 70) or neurological conditions affecting movement^1,44,45^. The study was double blind: experimenters did not know whether a participant was categorized into the rDCD or TD group at the time of testing, and participants and their parents did not know the hypotheses of the study. Participants of the CATCH study who were interested in participating in this study were identified as being in “group A” or “group B” by the CATCH research team. This allowed participants to be recruited in even numbers across the two groups, while our experimenters remained blind to group allocation during data collection. Three children were excluded from the analysis due to an additional diagnosis of autism spectrum disorder. Thus 43 participants were included in the analyses (n = 21 in rDCD). This research was reviewed and approved by the McMaster Research Ethics Board (MREB) #0411, and informed consent was obtained from both the parents/guardians and the participants. As of October 2020, parents/guardians signed an additional informed consent form outlining the lab’s procedure for testing with additional precautions due to COVID-19. All experiments and protocols were carried out in accordance with MREB ethical guidelines. The participants received a small toy, a certificate of participation, and a $40 gift certificate.

### General procedure

Participants performed the tasks in the following order: digit span test (~ 5 min), speech recognition with distorted timing task (~ 20 min), and tapping production tasks (~ 25–35 min), all in the same visit. The tapping tasks were always in the order of spontaneous motor tapping, metronome tapping, continuation tapping, and music tapping. Both groups (rDCD and TD) performed the experiment in the same order. Spontaneous motor tapping was performed first so that the tempi of the subsequent tasks would not influence the results. The MABC–2 and IQ were administered by the CATCH study prior to the date of the experiment (see “previously collected measures”).

### Speech recognition with distorted timing task

**Stimuli.** Participants were presented with speech stimuli through a pair of binaural Sennheiser headphones. Stimuli were sentences from the Coordinate Response Measure (CRM) corpus^47^. All sentences followed the same format: “Ready [call sign], go to [colour] [number] now.” Participants were asked to report the colour and the number they heard from a target sentence, which could always be identified with the call sign “Baron”. The target sentence was presented in a masking background of two sentences that were similar to the target sentence but used a different call sign. There were seven possible distractor call signs in the background sentences (“Eagle”, “Charlie”, “Tiger”, “Ringo”, “Hopper”, “Arrow”, and “Laker”). The target words were simple and highly familiar colours and numbers. Across the sentences, there were seven different possible target numbers (1–8, excluding seven as it has two syllables) and four possible target colours (“red”, “white”, “blue”, “green”). All conditions had two background talkers, with call signs, colours, and numbers that differed from the target sentence. Each trial consisted of one target sentence overlapped with two distractor background sentences. The target sentence with call sign “Baron” always used the same male voice, and the background sentences on each trial consisted of one female voice and one male voice. Background voices were randomly selected from six possible unique voices, which differed from the target voice. The two background sentences had onset asynchronies of − 50 and 50 ms, respectively, with respect to the onset of the target sentence. The three sentences together were presented at 55–60 SPL(A), and the signal-to-noise ratio between the target sentence and background sentences was always 2 dB.

In one condition block (intact speech rhythm), the rhythm of the target sentence was left intact. In the second condition block (modulated speech rhythm), the rhythm of the target sentence was modulated. The effect of this rhythm modulation was the key factor being investigated in this task, and the background sentences served as distraction in order to make the task optimally difficult for revealing potential differences. Modulation was applied by adjusting the rhythmic information of the target sentence in a sinusoidal pattern, causing the tempo to increase and decrease periodically within the same sentence. Rhythmic changes were applied using the Pitch Synchronous Overlap and Add (PSOLA) algorithm in Praat, which compressed and expanded the timing of the sentence using a compression ratio: CR(*t*) = 1 + m*sin(2πf_*m*_*t* + ϴ), where *m* equals the depth of modulation and f_*m*_ = 1 Hz^43^. In this experiment, *m* = 0.5 for the modulated speech condition. There were four possible phase shifts of the target modulation (π/4, 3π/4, 5π/4, and 7π/4), making the rhythm of the adjusted sentences unpredictable to the participants. Background sentences were always left intact.

**Procedure**. To begin, participants completed one block of 20 trials where the target sentence rhythm was modulated, but there were no background talkers. This was used to ensure participants were familiar with the modulated speech rhythms and were able to identify the target words in modulated speech when there were no distractor sentences. Participants then completed the two conditions (intact speech rhythm and modulated speech rhythm, both in the presence of distractor sentences), with two 20-trial blocks of each condition. The four condition blocks alternated between modulated and intact speech rhythm conditions, with initial block type counterbalanced across participants, for a total of 80 trials. Responses were considered correct when the participant reported both the colour and the number of the target sentence accurately. The only visual stimuli present during the task were buttons with all the possible colour and number choices. The participants provided their responses verbally, and the experimenter clicked the response button on the screen. The task was explained to participants in the context of a game: experimenters described a super spy mission to the children and said that they would be decoding secret messages.

### Tapping tasks

**Stimuli.** In all tapping task conditions, the stimuli were presented to the participants through a speaker in a sound attenuated room on average at 75.5 dB SPL(A) over a noise floor of approximately 28 dB SPL(A). A drum was placed on a table at a comfortable distance in front of the participant, and audio recordings of tapping data were collected through Audacity (version 2.1.0) at a sample rate of 8000 Hz. All auditory stimuli were 20 s in length. For the metronome and metronome-continuation tasks, sequences of percussive woodblock sounds (each 120 ms in duration) were presented at 400, 550, and 700 ms inter-onset intervals (IOIs). For the music excerpts, IOIs ranged from 500 to 700 ms, and the amplitude was ramped up and down over 500 ms at the beginning and end of each excerpt. The “true” beat onsets for each musical excerpt were determined by averaging the tapping data of two adult semi-professional drummers (for each excerpt, taps from nine trials were averaged, six trials from one drummer and three trials from the other), and the tempo of each excerpt was determined by the mean interval of beat onsets.

**Procedure.** Four different tapping tasks were adapted as a variation of the beat alignment test^48^. Participants were required to tap on a drum with their dominant hand while following prompts from the experimenter and visual signs on a screen indicating when to start and stop tapping. The visual signs included a green circle representing when the trial was in progress, and a red circle representing the end of a trial. Participants were allowed to talk to the experimenter between trials, and were motivated between trials by images of “Ziggy the Zebra” who gained stripes as the experiment progressed.

i. *Spontaneous Motor Tapping*. Participants tapped three trials of 20 s each at their preferred tempo. The experimenter instructed the participants to tap at a speed that was most comfortable to them, and to try to tap “steady like a clock”. Participants were allowed to practice a couple of times to find their preferred tempo before the trials began.
ii. *Metronome Tapping*. A metronome consisting of isochronous woodblock sounds was presented for participants to tap along to. Three different tempi (400, 550, and 700 ms IOIs) were presented for two 20 s trials each. The order of trials was randomized within the 6-trial block.
iii. *Metronome-Continuation Tapping*. On each 20 s trial, a metronome consisting of isochronous woodblock sounds was presented for 10 beats, at one of three different tempi (400, 550, and 700 ms IOIs). Participants synchronized their taps to the metronome, and after the 10 beats, participants were required to continue tapping at the same tempo for the remainder of the 20 s, without any external auditory cue. Two trials were completed at each tempo. The order of trials was randomized within the 6-trial block.
iv. *Music Excerpts*. Participants tapped along to the perceived beat of six different 20 s musical excerpts, chosen from pop and hip-hop genres (Table 1). Each excerpt was presented twice, and trials were randomized within the 12-excerpt block. To ensure participants understood the task was to tap to the beat and not the rhythm of the songs, before testing began, the experimenter sang “Happy Birthday” and had the participant tap along the beat. Before continuing with the testing, the experimenter ensured the participant understood how to tap the isochronous beat underlying the rhythm of the song and not the actual rhythm (which contained different event durations).

**Table 1:** Songs and tempi used in the music excerpt tapping condition.

Song title	Artist	Tempo (IOI)
Sing	Ed Sheeran	500 ms
Can’t Stop the Feeling	Justin Timberlake	530 ms
Yeah	Usher ft. Lil Jon	570 ms
Superstition	Stevie Wonder	600 ms
Love On Top	Beyoncé	638 ms
Umbrella	Rihanna ft. Jay-Z	690 ms

### Semi-professional musician ratings

For the music tapping conditions, a semi-professional percussionist (20 years of percussion experience, and 10 years of teaching experience) rated all trials on the following questions: (1) How synchronized was the tapping to the music, regardless of whether they tapped the beat or the rhythm?, (2) Is the participant always tapping the beat, or do they sometimes tap the rhythm?, (3) What percentage of the trial were they tapping the beat?, (4) When tapping the beat, how synchronized was the tapping to the music?, (5) When tapping the rhythm, how synchronized was the tapping to the music?, and (6) Was the tapping too quiet to hear? All questions were answered on a scale of 0–100. The percussionist was blind to the participants’ group allocations.

### Working memory task: digit span

The sum of the forward and reverse conditions of the digit span test from the Wechsler Intelligence Scale for Children, 4th Edition^49,50^ was used to measure participants’ working memory capacity.

### Questionnaires

Parents/guardians filled out two questionnaires: one regarding background in music, dance, and art; and the second the Conners’ Parent Rating Scale Revised, 27-item Short Version (CPRS-R:S) to assess for behaviours of ADHD. Children with T-scores 60 or above on the CPRS-R:S are considered to show atypical behaviour^51^.

### Previously collected measures

The MABC-2 was conducted by the INCH lab for all participants within one to two years prior to the collection of the data reported here. We used the sum of the aiming and catching, manual dexterity, and balance subtests. IQ scores were collected at the beginning of the INCH lab’s CATCH study using the Kaufman Brief Intelligence Test 2nd Edition (KBIT-2), when participants were five years old (two to four years prior to when the presented data were collected)^44^.

### Collection and analysis of tapping data

Tapping data were collected through Audacity in a WAV file format, time-locked with the auditory stimulus. We identified taps using an amplitude threshold identified individually for each participant, based on the intensity of their tapping. Each tap was then paired with the closest stimulus beat in time, and the distances from each tap to its associated beat were used to calculate circular statistics. Circular (or directional) statistics were used to calculate phase and consistency of tapping across metronome, continuation, and music trials. As shown in Fig. 2A, taps can be represented on a 360° polar scale, plotted by their relative angles from the metronome onset. The metronome/beat onsets are represented at angle 0, and the differences from 0 in tap times are measured in radians. A mean vector *R* was calculated by circularly averaging all taps from a single trial. The angle of vector *R* represents the mean phase, or the accuracy with which the participant tapped relative to the metronome/beat. Positive angles represent tapping after the onset of the metronome/beat, and negative angles tapping before. The length of *R* represents consistency, which can be thought of as the reciprocal of variability. Values range between 0 and 1, where 1 represents perfect consistency (i.e., the asynchrony between every tap and metronome/beat onset is identical across every tap in the trial), and 0 represents a circular-uniform random distribution of tap times relative to the beat. Consistency data was submitted to a logit transformation before further analysis to reduce data skewness, as is common in tapping synchronization research^52,53^.

### Ethical approval

This research was reviewed and approved by the McMaster Research Ethics Board (MREB).

## Results

### Demographics

43 children between the ages of 7 and 10 years were tested (Table 2). The rDCD group (n = 21) averaged in the 10th percentile on the MABC-2 (SD = 4.9), ranging from the 0.5th–16th percentile, while the TD group (n = 22) averaged in the 53rd percentile (SD = 24.2), ranging from the 25th–95th percentile (see Figure S1 for a histogram of MABC-2 percentiles). Performance on the digit span task did not differ significantly between groups (*t*(40.1) = 1.11, *p* = 0.273). There was no significant difference between groups on the CPRS-R:S (*t*(35.2) = 1.86, *p* = 0.072). Six participants had T-scores > 60, which fall in the range of atypical behaviour; however, they were spread across the rDCD (four) and TD (two) groups and none of these participants reported a diagnosis of ADHD. This questionnaire is intended to be interpreted by a trained medical doctor in combination with teacher reported questionnaires and further interviews and assessments. Further, four of the six children were female and the CPRS-R:S requires higher raw scores for female participants to be assigned a T-score over 60^51^. Given these factors, we did not exclude participants with T-scores > 60, but we have identified them in all figures.

**Table 2:** Participant demographics of study sample in rDCD and TD groups.

Variable	rDCD (± SD)	TD (± SD)	T-Test *P* Value
Sample size	21	22	N/A
Females:Males	4:17	13:9	N/A
Age	8.9 ± 0.7	8.8 ± 0.8	.861
MABC-2 Percentile	10 ± 4.9	53 ± 24.8	< .001
Digit Span ^†^	16.3 ± 3.3	15.2 ± 3.0	.273
Years of music training	1.38 ± 1.8	1.41 ± 1.6	.957
CPRS-R:S ^‡^	54.1 ± 8.3	50.1 ± 5.7	.072
SES ^§^	4.2 ± 1.4	5.0 ± 2.0	.131

^†^Maximum digit span score = 32.

^‡^Conner’s Parent Rating Scale Revised (Short): T-scores > 60 represent atypical behaviour.

^§^SES (socioeconomic status) was estimated by average household income per year, scored on a scale of 1–7, where 1 =  < $30,000, 2 = $30,000–60,000, 3 = $60,000–90,000, 4 = $90,000–120,000, 5 = $120,000–150,000, and 6 =  > $150,000.

The two groups did not differ in their years of music training, *t*(39.4) = -0.05, *p* = 0.957. The ratio of male to female participants in the rDCD group was large (only 4 of the 21 participants were female) compared to the TD group (13 female out of 22 participants). This is not unusual for studies involving children with DCD, as reports of the ratio of males to females who are diagnosed with DCD range from 2:1 to 7:1^54^. No significant differences between male and female participants were found for any main outcome variables in the TD group (see supplementary data for details). Age was used as a covariate for analyses in both the speech recognition with distorted timing task and the tapping tasks, as tapping synchronization and beat perception change significantly across the 7–10 year age groups^55^, and age has been found to be a significant factor in previous studies using the same age range^56^.

## Speech recognition with distorted timing task

### Proportion of correct responses

**Figure 1:**
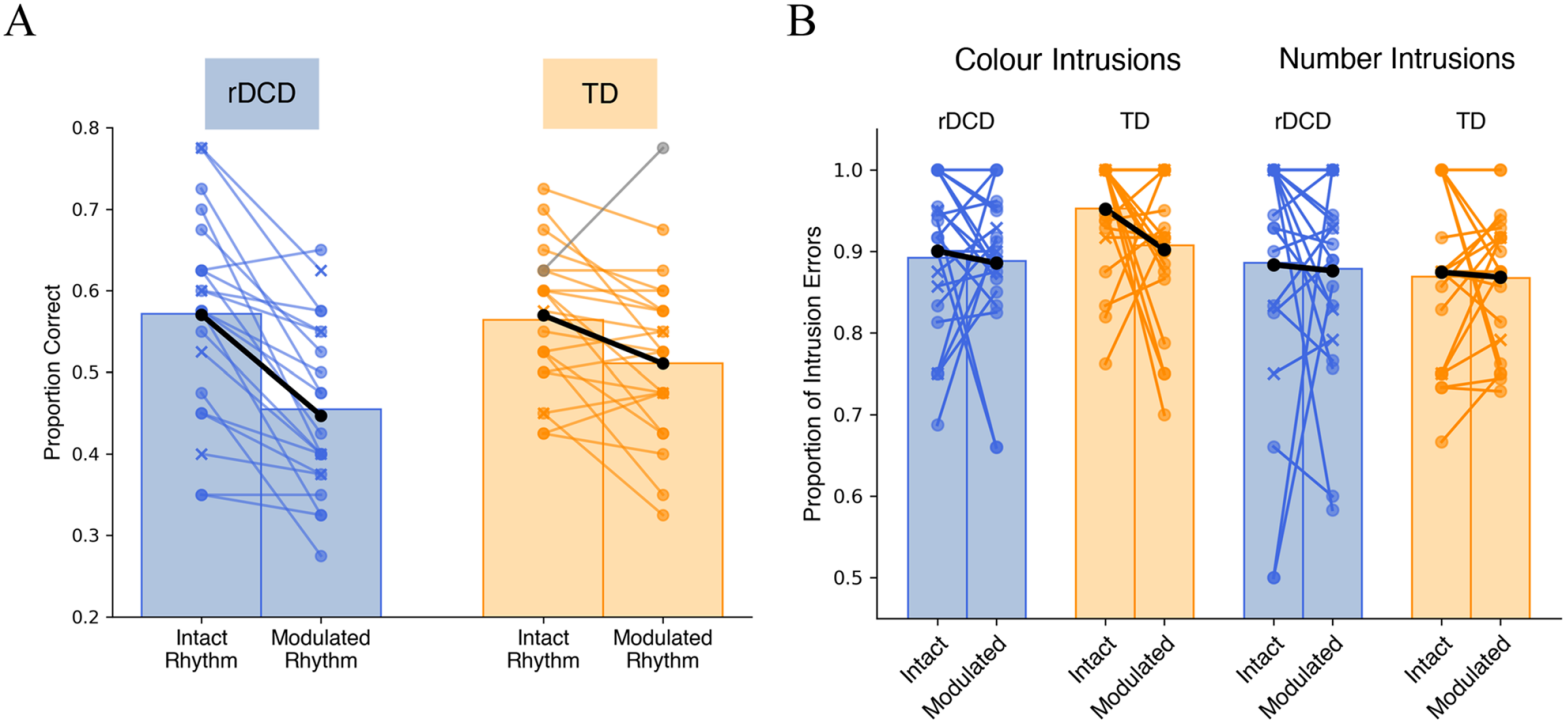
Mean proportion of correct responses and intrusion errors on the speech recognition with distorted timing task. rDCD (blue) and TD (orange) with group means represented in black. Participants with CPRS-R:S T-scores > 60 are represented by Xs. (**A**) Mean performance and individual data across conditions for intact rhythm and modulated rhythm conditions (each dot represents one participant, with lines connecting data from the same participant, and an outlier in grey) showing a significant interaction of group and condition (*p* = 0.017). (**B**) Intrusion errors (trials where the incorrect responses were words from one of the two background sentences) as mean proportions of incorrect responses, showing a significant group effect for colour intrusions (*p* = 0.040), but not number intrusions (*p* = 0.653).

Proportion of correct colour and number word recognition was calculated for the speech recognition with distorted timing task by dividing the number of trials where both the colour and the number were correctly identified by the total number of trials per condition. In the first block with modulated rhythms and no background talkers, both groups had near perfect scores (rDCD: M = 0.99, SD = 0.01; TD: M = 0.99, SD = 0.02). For the intact and modulated speech conditions, mean proportion of correct scores across groups are summarized in Fig. 1A. One outlier in the TD group was removed, as it exceeded the upper inner fence of the 1.5 interquartile range (a common threshold for identifying outliers^57^) of responses in the modulation condition. A 2 × 2 mixed-design analysis of variance (ANOVA) was conducted to compare the within-subject variable of rhythm condition (intact vs. modulated speech rhythm) and the between-subject variable of participant group (rDCD vs. TD) with age as a covariate. Data was found to be normally distributed, assessed by the Shapiro–Wilk test, and sphericity assumptions were met. Results revealed a significant interaction between group and condition, *F*(1,39) = 6.20, *p* = 0.017, η^2^ = 0.023. The group effect was non-significant, *F*(1,39) = 0.69, *p* = 0.410, η^2^ = 0.014, as was the effect of condition, *F*(1,39) = 0.002, *p* = 0.963, η^2^ < 0.001. To investigate this interaction, a post-hoc paired t-test showed a significant difference between proportion of correct responses in the intact compared to modulation conditions for the rDCD group alone, *t*(20) = 5.86, *p* = 0.005, *d* = 1.28 (Bonferroni corrected). To further investigate, a difference score was calculated for each participant by their average performance on the modulated rhythm condition subtracted from their average performance on the intact rhythm condition. A t-test (Bonferroni corrected) showed a significant difference between groups for difference scores (*t*(37.5) = 2.51, *p* = 0.033, *d* = 0.78), with the rDCD group (M = 0.12, SD = 0.09) showing a larger difference between conditions than the TD group (M = 0.05, SD = 0.07).

### Intrusion errors as a proportion of incorrect responses

Intrusion errors were defined as trials where the participants’ responses were words from one of the two background sentences instead of the target sentence. These trials reflect poor attention at the critical locations in time^42^. A 2 × 2 mixed-design ANOVA on proportion of colour intrusions revealed a significant effect of group, *F*(1,40) = 4.52, *p* = 0.040, η^2^ = 0.05; although the TD group had fewer colour response errors, a higher proportion of these errors were intrusions compared to the rDCD group (Fig. 1B). There was no effect of condition, *F*(1,40) = 1.22, *p* = 0.276, η^2^ = 0.014, or interaction*.* For number intrusions, there were no effects of group, *F*(1,40) = 0.20, *p* = 0.653, η^2^ = 0.003, condition, *F*(1,40) = 0.26, *p* = 0.611, η^2^ = 0.003, or interaction (Fig. 1B)*.*

## Tapping production tasks

### Spontaneous motor tapping

Spontaneous motor tapping tempos were defined as the median IOI averaged across the three spontaneous tapping trials. Within each trial, tap intervals were removed if they were identified as outliers by the interquartile range method. Although the rDCD group (M = 806 ms IOI, SD = 297) tended to tap faster than the TD group (M = 860 ms IOI, SD = 296), an independent samples t-test revealed no significant difference between the two groups, *t*(41) = -0.60, *p* = 0.553, *d* = − 0.18 (Figure S2A). Tapping variability was defined by the coefficient of variation of the IOI (SD of IOIs/mean IOI). The rDCD group (M = 0.06, SD = 0.007) had a similar variability to the TD group (M = 0.06, SD = 0.05), and the difference was non-significant, *t*(38.1) = 0.12, *p* = 0.905, *d* = 0.04 (Figure S2B).

### Analyses of metronome, music and continuation tapping

Trials were excluded from analysis that were identified by the semi-professional musician rater as “rhythm tapping”, in which the participant tapped the rhythm of the musical excerpt rather than the beat. In the rDCD group, 35 out of 252 trials were identified as the participant tapping a rhythm as opposed to the beat and 13 of the 21 participants had at least one trial of rhythm tapping. In the TD group, only 15 out of 264 trials were identified as rhythm tapping, and only 5 of the 22 participants had at least one trial of rhythm tapping. These proportions were significantly different between groups, *X*^2^(1) = 14.97, *p* < 0.001. The pattern of results remained the same if these ‘rhythm tapping’ trials remained in the analysis (see supplementary data).

In the metronome condition, all taps were analyzed. In the metronome-continuation condition, only taps during the continuation phase were analyzed (i.e., taps during the initial 10 metronome beats were excluded), and taps were analyzed relative to where the metronome would have been if the auditory cue had continued. To ensure results weren’t skewed by one group speeding up or slowing down more than the other group, linear regression lines for each continuation trial were calculated using the tapping intervals (Figure S3). A repeated measures ANOVA showed no difference in regression slopes between groups (*F*(1,41) = 0.01, *p* = 0.907, η^2^ < 0.001), but there was a significant effect of tempo (*F*(1.4, 57.5) = 6.49, *p* = 0.007, η^2^ = 0.086). Slopes during the 700 ms IOI tempo differed significantly from both the 550 ms tempo, *t*(42) = 2.92, *p*_*corr*_ = 0.017, *d* = 0.60, and the 400 ms tempo, *t*(42) = 4.11, *p*_*corr*_ < 0.001, *d* = 0.84 (Bonferroni corrected), indicating that participants sped up more during the slowest tempo. There was no significant interaction between group and tempo (*F*(1.4, 57.5) = 1.61, *p* = 0.213, η^2^ = 0.02). In metronome and continuation conditions, trials were averaged across the three tempi (400, 550, and 700 ms IOI), as no effect of tempo was found in either consistency or phase accuracy. For detailed analyses of consistency and phase scores with tempo as a factor, see supplementary data (Figure S4). For the tapping data, the assumption of sphericity was violated, such that the variances of the differences between conditions were not homogeneous (*X*^*2*^ = 10.23, *p* = 0.006 for consistency, *X*^*2*^ = 7.55, *p* = 0.023 for phase). Degrees of freedom for effects of condition and its interactions were corrected using Greenhouse–Geisser estimates (ε = 0.813 for consistency, ε = 0.850 for phase). Data was found to be normally distributed, as assessed by the Shapiro–Wilk test.

**Figure 2:**
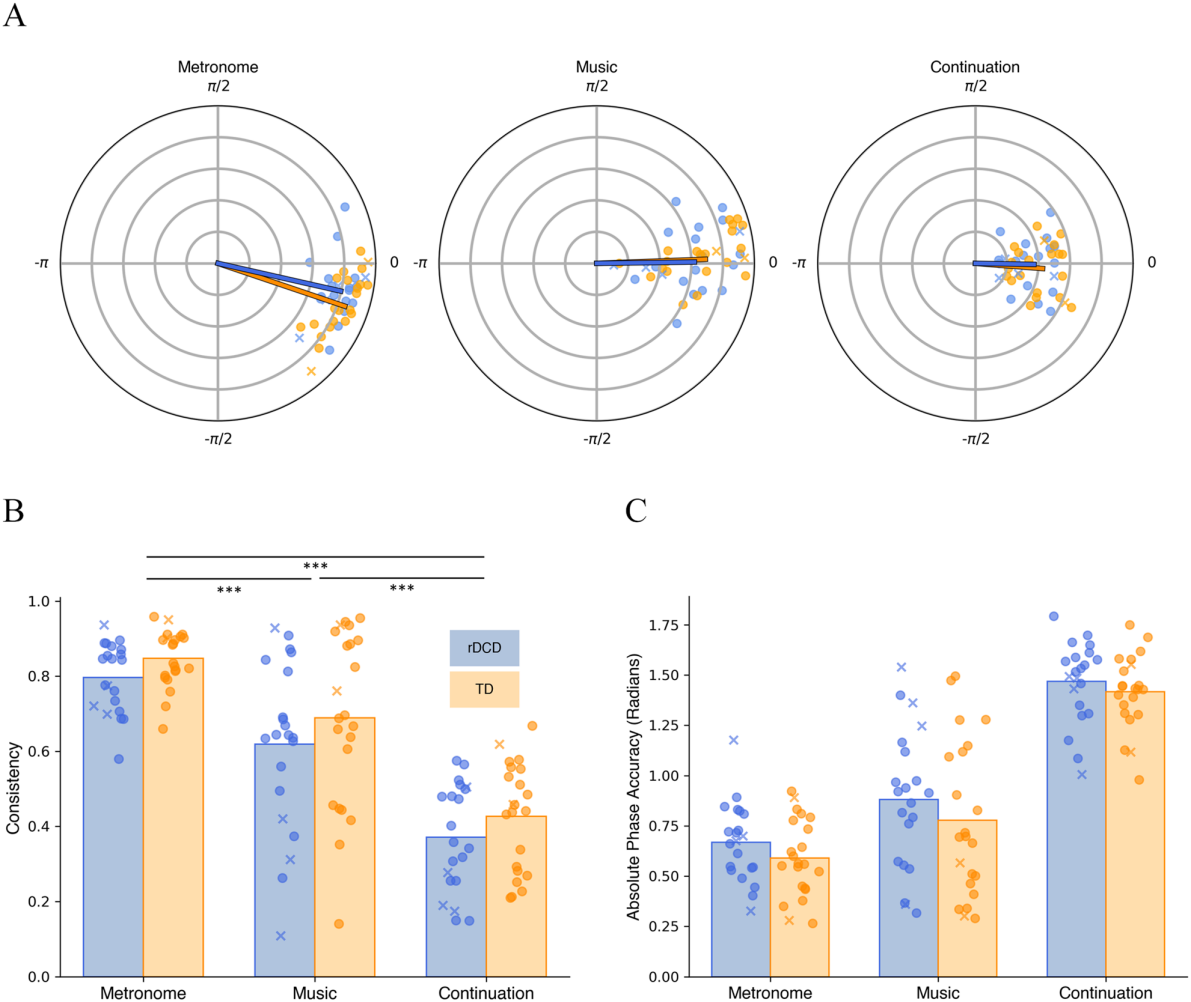
Consistency and phase accuracy scores for rDCD and TD groups in each tapping condition, averaged across tempi*.* Dots show individual data for rDCD (blue) and TD (orange) groups. Participants with CPRS-R:S T-scores > 60 are represented by Xs. (**A**) Polar plots in which tapping consistency is represented by the length of the mean vector *R* and accuracy is reflected by the angle (phase) of the mean vector *R*, measured in radians. The time of the stimulus beats is at 0. (**B)** Consistency scores in tapping production tasks showing significant effects of group (*p* = 0.041) and condition (*p* = 0.007). (**C**) Absolute value of tapping accuracy scores (radians) in tapping production tasks show a significant effect of condition (*p* = 0.032). * *p*_*corr*_ < 0.05; ** *p*_*corr*_ < 0.01; *** *p*_*corr*_ < 0.001 (Bonferroni corrected).

Tapping consistency for the musical excerpts was calculated similarly as for the metronome and continuation data, but with the stimulus beat locations defined by the tapping of the semi-professional musicians (see “Methods”). Music tapping trials were averaged across all six songs (for data separated by song, see Figure S5). An ANOVA with factors condition (metronome, music, and continuation) and group (rDCD, TD) and age as a covariate showed a significant effect of group, *F*(1,40) = 4.46, *p* = 0.041, η^2^ = 0.045*,* and a significant effect of condition, *F*(1.63, 65) = 6.05, *p* = 0.007, η^2^ = 0.053 (Fig. 2B), but no interaction of group and condition, *F*(1.63, 65) = 0.29, *p* = 0.702 η^2^ = 0.003. To further explore whether both metronome and music tapping (conditions with an auditory cue) had higher consistency than continuation tapping (condition without an auditory cue) in the rDCD alone, t-tests (Bonferroni corrected) were used to compare each pairwise combination of conditions in the rDCD group. Metronome tapping was significantly more consistent than both music tapping (*t*(20) = 4.20, *p* = 0.001, *d* = 0.92) and continuation tapping (*t*(20) = 12.0, *p* < 0.001, *d* = 2.62), and music tapping was more consistent than continuation tapping (*t*(20) = 5.89, *p* < 0.001, *d* = 1.28). Similar to the speech recognition task, a t-test examined difference scores between consistency when the beat was present (metronome) and when it was it was removed (continuation), and showed no difference between groups, *t*(40.8) = 0.46, *p* = 0.645, *d* = 0.14.

Phase accuracy (vector angle), analyzed using a 2 × 3 mixed design ANOVA with age as a covariate, revealed no significant effects of condition (*F*(1.7,68) = 0.19, *p* = 0.796, η^2^ = 0.003), group (*F*(1,40) = 0.57, *p* = 0.457, η^2^ = 0.006), or interaction of group and condition (*F*(1.7,68) = 0.55, *p* = 0.552. η^2^ = 0.008) (Fig. 2A). To investigate the absolute angular offset regardless of direction (tapping before vs. after the beat), we calculated the absolute value of the distance of taps from the beat in radians. A 2 × 3 mixed design ANOVA with age as a covariate found a significant effect of condition (*F*(1.8,72.6) = 3.60, *p* = 0.037, η^2^ = 0.040), but no significant effect of group (*F*(1,40) = 2.19, *p* = 0.146, η^2^ = 0.023) or interaction of group and condition (*F*(1.8,72.6) = 0.17, *p* = 0.823, η^2^ = 0.002) (Fig. 2C).

### Relations among motor skills, speech recognition with distorted timing conditions and tapping consistency

**Figure 3:**
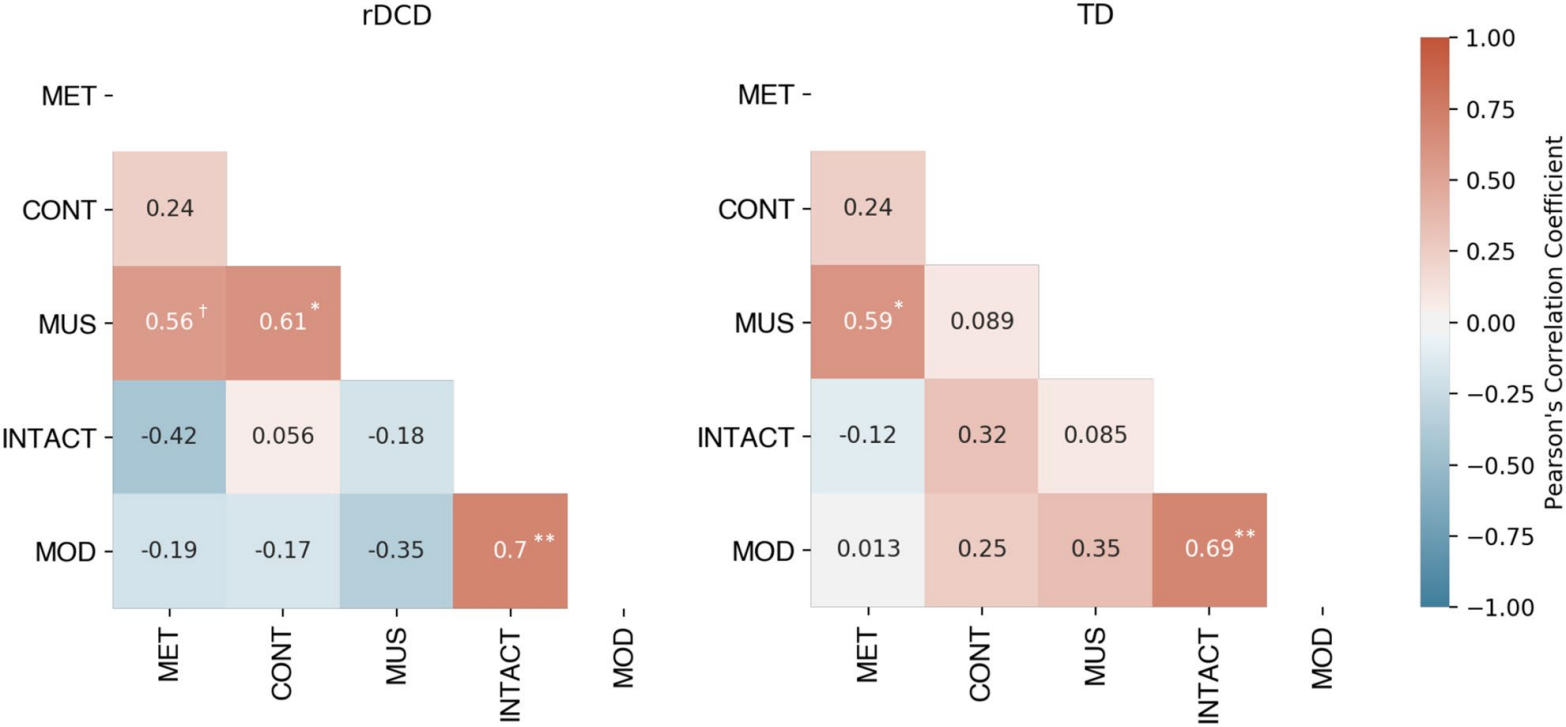
Heatmap of Pearson’s correlation coefficients for both rDCD and TD groups. MET = Metronome Tapping; CONT = Continuation Tapping; MUS = Music Tapping; INTACT = Intact Speech Rhythm Recognition; MOD = Modulated Speech Rhythm Recognition. * *p*_*corr*_ < 0.05; ** *p*_*corr*_ < 0.01; *** *p*_*corr*_ < 0.001; † *p*_*corr*_ < 0.08 (Holm’s corrected).

To investigate relations among the five tasks in the present study, Pearson’s correlation coefficients were calculated for each pair of conditions (accuracy scores for the two speech recognition conditions [with and without modulated timing] and consistency scores for the three tapping conditions). Correlations for tapping conditions were conducted on logit-transformed consistency scores. For speech recognition with distorted timing conditions, correlations were conducted on proportion of correct responses. In the rDCD group, the three tapping conditions had correlation coefficients in the positive direction (metronome to continuation: *r(19)* = 0.24, *p*_*corr*_ > 0.999; metronome to music: *r(19)* = 0.56, *p*_*corr*_ = 0.071; continuation to music: *r(19)* = 0.61, *p*_*corr*_ = 0.030). The two speech recognition with distorted timing conditions were highly correlated, *r(19)* = 0.70, *p*_*corr*_ = 0.004, and both speech recognition conditions had negative or close to zero correlation coefficients with the tapping tasks, with none reaching significance. The TD group showed similar results, with correlation coefficients in the positive direction for metronome tapping with continuation and music tapping (metronome to continuation: *r(20)* = 0.27, *p*_*corr*_ > 0.999; metronome to music: *r(20)* = 0.64, *p*_*corr*_ = 0.015), although continuation and music tapping showed no relation (*r(20)* = 0.04, *p*_*corr*_ > 0.999). Again, the two speech recognition with (and without) distorted timing conditions were highly correlated (*r(20)* = 0.69, *p*_*corr*_ = 0.005), and showed a mix of positive and negative correlations to the three tapping conditions, but none reached significance. All p-values were corrected for multiple comparisons by Holm’s method, and all correlations are summarized in Fig. 3 and plotted in Figure S6.

To explore the relations between motor skills and the timing tasks of the present study, Pearson’s correlations were calculated between MABC-2 scores and (1) modulated speech recognition accuracy scores (of interest as rDCD and TD groups differed on this measure) and (2) general tapping performance (“tapping scores” were calculated as the average of consistency scores in metronome, continuation, and music tapping conditions). Given that the rDCD and TD children came from the same sample of children and the MABC-2 scores are a continuous measure ranging from the 1st–95th percentile, it is justified to include both groups together in an exploratory correlational analysis (Figure S7). Performance on the modulation condition of the speech recognition with distorted timing task correlated significantly with MABC-2 scores (*r(40)* = 0.37, *p*_*corr*_ = 0.033). There was also a trend for tapping consistency to correlate with MABC-2 scores, *r(40)* = 0.29, *p*_*corr*_ = 0.053, but it did not reach significance after correcting for multiple comparisons (p-values corrected by Holm’s method).

## Discussion

The present study showed that children with rDCD can use rhythmic tracking to benefit their performance during speech recognition and auditory-motor synchronization. Children with rDCD showed deficits in identifying target words in the presence of distractor sentences when speech is rhythmically modulated, although their performance was indistinguishable from TD children when the sentences were normally-timed. Children with rDCD also tapped to a beat less consistently than TD children, but (like TD children) tapped with significantly higher consistency when an auditory cue (i.e., a metronome or music) was present compared to when there was no auditory cue. As the multimodal integration of auditory-motor synchronization of DCD has rarely been investigated, our findings provide a novel connection between deficits in auditory timing perception^25^ and on repetitive motor tasks^12^ in DCD. These findings imply that research on task-oriented interventions for children with DCD may include an auditory rhythmic cue, as the presence of an auditory cue may help to guide the timing of the motor skill during training, which in turn may provide additional benefit compared to motor-only therapeutic strategies^58^.

### Children with rDCD rely on rhythmic cues in speech recognition more than TD children

Both groups were able to identify target words in the presence of distractor sentences with similar accuracy when the rhythm of the sentences was left intact. However, when the rhythm of the sentences was modulated, the rDCD group’s recognition of target words suffered more, suggesting that, in comparison to TD children, those with rDCD were more reliant on the familiar rhythmic cues in language and less flexible in adapting to modulated speech rhythms. The lack of a difference between rDCD and TD children for the rhythmically intact sentences appears, at first glance, at odds with the previous finding that children at risk for DCD had higher thresholds for detecting small timing deviations in isochronous metronomes^25^. However, the two tasks differ in that our speech task requires using rhythmic tracking to detect speech content whereas the previous task of detecting timing deviations in a metronome requires retrospective evaluation of the rhythm itself. Another major difference is that children must recognize speech with intact rhythms every day of their lives, whereas metronome listening is not part of normal life, and our results indicate that children with rDCD are well able to use rhythmic cues to decipher speech. However, the fact that children with rDCD exhibit greater performance decline than TD children when speech rhythm was modulated implies less flexible adaptability in their rhythm perception. This is consistent with previous visual-motor work showing children with DCD are slower to adapt to changes in visual information^43^. To our knowledge, this is the first study to show the impact of poor adaptation to modulated auditory rhythmic timing in a context that does not involve an overt motor component.

Although the rDCD group had more colour name errors overall, the TD group displayed a higher proportion of intrusion errors from the background (to be ignored) sentences, whereas children with rDCD were more likely to guess a random colour, suggesting the TD group displayed greater misdirected attention while the rDCD group displayed a more general distraction associated with the modulated speech rhythms.

### Auditory-motor synchronization in DCD

Being able to perform movements at specific times and in rhythmic patterns are important skills in the development of motor abilities, as many everyday motor tasks involve predicting and executing specific timing. Children with rDCD showed significantly less consistent tapping across metronome, continuation, and music conditions than TD children. However, importantly, both groups had significantly greater tapping consistency (in relation to the beat or implied beat) in conditions with an auditory cue (metronome and music) than without (continuation tapping). This performance benefit of having the auditory cue present was shown similarly in both groups, as evidenced by the non-significant difference between groups in the difference scores for consistency between metronome and continuation. This shows that children with rDCD can rhythmically track auditory cues, integrate the auditory cues and motor commands, and the auditory cues help them to perform more consistent movements. More specifically, performance was more consistent for tapping to the metronome than to music for both rDCD and TD groups. This is perhaps not surprising as the beat is directly given by the metronome, but must be extracted from the complex acoustic signals of music. Still, children with rDCD benefitted and showed more consistent tapping to music than in the continuation condition where auditory cues were absent.

For phase, both groups performed very similarly to one another across conditions, suggesting both groups were able to accurately track the beat of the stimuli. This is similar to Lê et al.’s (2021) finding that children with DCD had similar accuracy to TD children when synchronizing a verbal response to an isochronous rhythm^17^. When tapping to an auditory stimulus, adults typically tap ahead of the beat^52,59^. Consistent with this, in the metronome condition, children showed a mean negative asynchrony, demonstrating the operation of predictive processes for when they expected the next beat. On the other hand, mean asynchronies for continuation and music tapping were close to zero, with some participants tapping before the beat, and some tapping after. It is likely that this reflects that the children sometimes anticipated the beat, as with metronome tapping, while sometimes reacted to the beat, and hence tapped on the late side, as the absolute values of phase differences between taps and stimuli show the metronome to have the least absolute asynchrony, followed by the music, followed by continuation trials. In any case, the trends across conditions are similar to studies in adults that have found metronome tapping to have a larger negative asynchrony than music tapping^52,60^. Most importantly, children with rDCD showed very similar mean phase alignment to the beat as TD children across all the conditions indicating that they are able to coordinate motor tapping to auditory cues.

While phase alignment was similar across the groups, it is interesting that in the music conditions, a significantly higher proportion of the rDCD group tapped the rhythm of the music as opposed to the beat, compared to the TD group. This group difference was seen not only in the total proportion of music tapping trials, which could be driven by a few participants tapping the rhythm to all of the songs, but also in the number of participants who had at least one trial of rhythm tapping. As mentioned above, unlike with metronome tapping for which every auditory sound is a beat, tapping to music requires the participant to extract the steady isochronous beat from multiple cues in the music, including different note durations (e.g., quarter notes, eighth notes, etc.), pitch and harmonic cues to beats, and multiple instruments playing simultaneously. The difference between groups in the frequency and number of children who tapped the rhythm rather than the beat in the music condition aligns with our interpretation of the speech perception results, in which the rDCD group was less adaptable to changes in what they expected the speech timing to be. This effect is unlikely to be due to a misunderstanding of the task, as the experimenter ran a practice trial with the participants to ensure they understood what the “beat” of the song was, and reminded participants between trials to tap the beat if they noticed any rhythm tapping. It is possible that the rDCD group had more difficulties with inhibition (i.e. inhibiting their desire to tap the rhythm), increased cognitive demands (i.e. extracting the beat of the music instead of tapping the more obvious rhythmic pattern), or an increase in distraction by the rhythm.

In the spontaneous motor tempo task, both groups showed slower tapping than has been reported previously in the literature. For example, McAuley et al. (2006) reported that children of this age prefer to tap around 520 ms intervals^55^, whereas we found mean tapping intervals to be 806 ms and 860 ms for the rDCD and TD groups, respectively. We believe this difference may have arisen from our instruction, in which we asked the participants to tap, “steady like a clock,” in an effort to explain that the goal of the task was to tap as consistently as possible. However, this may have led the participants to attempt to match the timing of a clock, thus influencing them to tap slower than they otherwise would have.

### Relations among motor skills, speech conditions and tapping consistency

Previous studies in TD adults show a positive correlation between perception and production skills for auditory timing and auditory-motor synchronization^48,52,61,62^. In the present study, perceptual and perceptual-motor consistency was not significantly correlated. This might be because we used a speech task for our perceptual measure, but metronome and music tasks for auditory-motor synchronization. It is also worth noting that our speech task was not a beat-based task, whereas the tapping tasks were, which may affect the relation between the two. As well, our speech task also cannot be directly compared directly to Chang et al.’s (2021) finding of auditory perceptual timing deficits in children with rDCD. Not only did they use rhythm and duration discrimination tasks rather than speech-based tasks, but the nature of the tasks was different, as our speech recognition with distorted timing task did not require the participants to make judgments about speech timing. Our speech task also contained an auditory distraction component, which Chang et al.’s (2021) task did not^25^. A possible interpretation of these null correlational findings is that music and speech tasks may be tapping into somewhat different underlying processes.  Future work should explore the relation between auditory-motor synchronization and perceptual beat perception using more relatable tasks.

We also did an exploratory investigation on whether an overall measure of auditory motor synchronization (tapping consistency on the metronome, continuation, and music tapping tasks) and performance on the rhythmically modulated speech perception task were correlated with the MABC-2 percentiles. Though most of the analyses in this study used group (rDCD or TD) as a factor, as group differences were the main focus of this research, using the MABC-2 percentiles as a continuous measure allowed a broader investigation into the relation of general motor skills and our tasks. Both tapping consistency and modulated speech recognition had positive correlations with the MABC-2. We did not have the sample size to examine these correlations in the groups separately, so more research is necessary to fully understand the relationship between auditory timing perception and general motor skills.

### Rhythm deficits in neurodevelopmental disorders

Our study excluded participants with autism spectrum disorder, but we did not assess for dyslexia or developmental language disorder. While this can be seen as a limitation, DCD is reported to be highly comorbid and overlap in symptoms with other developmental disorders^1,29,63^, so attempting to identify groups of participants with “pure” DCD would not reflect the true heterogenous nature of the group. We did, however, exclude participants with autism spectrum disorder, as there is some debate as to whether children with ASD can have an additional diagnosis of DCD^64,65^. Beyond DCD, evidence is accumulating that timing and rhythm deficits are associated with many neurodevelopmental disorders, including dyslexia, attention deficits and autism spectrum disorder^29,66^. Our findings of impaired synchronized tapping production, along with Chang et al.’s (2021) findings of impaired rhythm and duration perception fit in well with these studies^25^. However, as noted by Lense et al.’s (2021) review, there is not a clear or linear relation between neurodevelopmental disorders and associated rhythm deficits^63^. More research is needed to further understand individual differences in DCD, and how they relate to rhythm and beat perception and production.

## Conclusion

Compared to age-matched typically developing children, children at risk for DCD were found to be significantly less consistent during auditory-motor synchronization tapping tasks, as well as more reliant on intact rhythms during a speech recognition task involving no overt motor component. At the same time, motor tapping performance in children with rDCD showed similar benefit as for TD children with the presence of an auditory cue. These results fit in well with research showing that neurodevelopmental disorders that are highly comorbid with DCD, such as attention deficits and dyslexia, also have associated deficits in auditory timing perception and auditory-motor tapping consistency. Given that children in both the rDCD and TD groups had more consistent tapping when an auditory cue was present, it is worth investigating interventions for children with DCD that combine rhythmic auditory stimuli and repetitive movements.

### Supplementary Information

Supplementary Information.

## Data Availability

The data that support the findings of this study are available on request from the corresponding author.
