# Supplementary material for: Auditory rhythm facilitates perception and action in children at risk for developmental coordination disorder
Source: Sci Rep. 2024 May 28;14:12203. doi: 10.1038/s41598-024-62322-6 (PMC11133375; doi:10.1038/s41598-024-62322-6)
Supplement: Supplementary file 1 — Supplementary Information. [file 41598_2024_62322_MOESM1_ESM.pdf]

Auditory rhythm facilitates perception and action in children at risk for Developmental Coordination Disorder

Authors: Chantal Carrillo, Andrew Chang, Hannah Armstrong, John Cairney, J. Devin McAuley, and Laurel J. Trainor

Supplementary Material

Movement Assessment Battery For Children – 2nd Edition

The Movement Assessment Battery for Children - 2nd Edition (MABC-2) [1] was used as the standardized measure of motor coordination. Individually administered raw scores were converted into standard scores based on the child's age, and then converted into an overall percentile. Test-retest reliability and standard error of measurement for the total test scores are reported to be 0.80 and 1.34, respectively [1]. All participants were in the same age band, and thus were all tested on the same installment of the MABC-2 (Age Band 2). The MABC-2 data was collected as part of the CATCH study [2,3], on average 11.15 ± 6.03 months prior to participating in the experiment. Participants' MABC-2 scores ranged from the 0.5th-95th percentile. Children scoring at or below the 16th percentile were placed in the probable developmental coordination disorder (rDCD) group, and children above the 16th percentile were in the typically developing (TD) group (Figure S1).

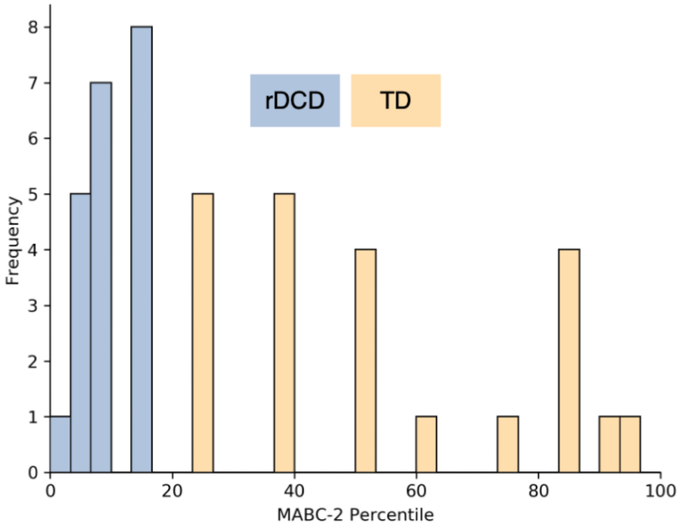

Figure S1. Histogram of MABC-2 Percentile Scores.

The Effect of Sex on Speech Recognition and Tapping Production Consistency

Sex was not included as a covariate in any of the main analyses due to the low number of female participants in the rDCD group (four out of 21 participants). To ensure sex was not a significant predictor of major outcome variables, t-tests between male and female participants were conducted for the TD group alone, as the TD group has a much more even distribution of boys and girls (13 of 22 participants were female). A Welch's t-test of overall tapping score

(mean consistency for metronome, continuation, and music tapping) found no significant difference between boys and girls, $t(19.8) = 0.03$, $p = .976$, $d = 0.01$. A Welch's t-test of the difference score in the speech recognition task (difference in proportion of correct responses between intact and modulated conditions) also found no significant difference between boys and girls, $t(14.7) = 0.65$, $p = .524$, $d = 0.29$.

Investigating Differences Between Groups During Spontaneous Motor Tempo Tapping

T-tests comparing group differences during spontaneous motor tempo tapping revealed no significant differences in preferred tempo ($t(41) = -0.60$, $p = .553$, $d = -0.18$) or coefficient of variation ($t(41) = 0.12$, $p = .905$, $d = 0.04$). Coefficient of variation was defined as the standard deviation of intervals divided by the mean interval across a trial. Individual data is shown in Figure S2.

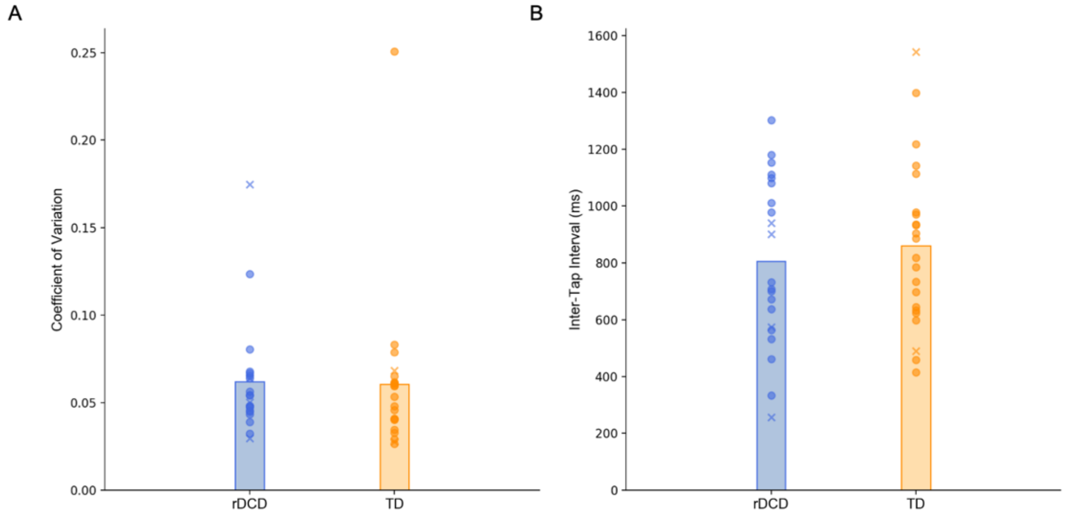

Figure S2. Spontaneous motor tempo tapping. Participants with CPRS-R:S T-scores >60 are represented by Xs. (A) Mean inter-onset intervals for both rDCD and TD. (B) Mean coefficient of variation of inter-onset intervals. Dots represent individual data.

Investigating Whether Participants Sped Up or Slowed Down During Continuation Tapping

During continuation tapping, consistency and phase scores were calculated using the difference in time between where the participants tapped and where the auditory metronome would have been, had it continued. To ensure these results weren't being skewed by one group slowing up or speeding down over the course of a trial more than another group, we ran a linear regression analysis on the inter-tap intervals of each trial for each participant. The slope of the calculated regression lines were compared between groups and tempi in a 2x2 mixed ANOVA, and no effect of group was found ($F(1,41) = 0.01$, $p = .907$, $\eta^2 < .001$). A significant effect of tempo was observed ($F(1.4, 57.5) = 6.49$, $p = .007$, $\eta^2 = .086$) in which the slowest tempo (700 ms IOI) had the greatest amount of change in speed, and the fastest tempo (400 ms IOI) had the least. Mean regression lines are shown in Figure S3.

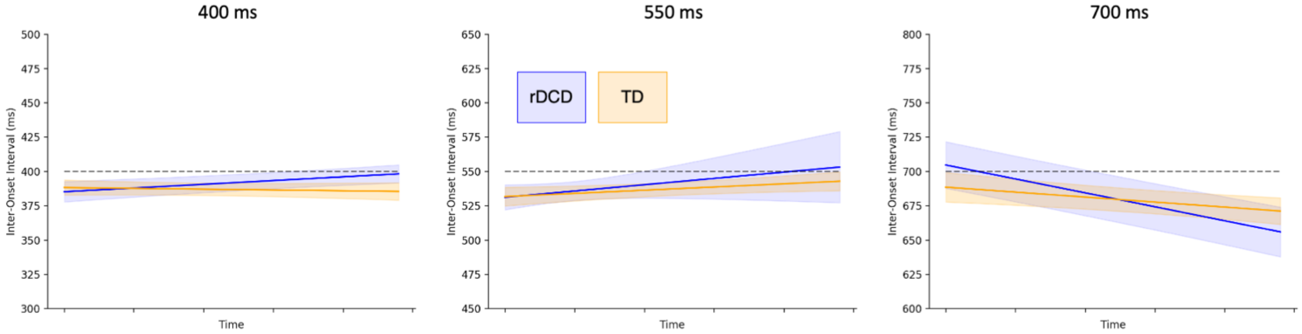

Figure S3. Mean regression lines for inter-tap intervals during continuation tapping, representing participants speeding up or slowing down their tapping speed across a trial. The grey dotted line is at the stimulus tempo, thus represents what the regression line would look like if participants tapped at the correct speed with no speeding up or slowing down (slope = 0).

The Influence of Tempo on Tapping Production Consistency

Consistency scores during tapping production tasks were calculated using circular statistics to find the length of the average vector *R* (Figure S4A). Scores range from 0-1, and were submitted to a logit transformation before further analysis. Consistency scores were first compared between the metronome and continuation conditions (excluding the music condition), as the tempi in these two conditions were matched (Figure S4B). For both conditions, there were two trials per tempo, and these were averaged prior to conducting the statistical analysis. All sphericity and normality assumptions were met. A 2x2x3 mixed-design ANOVA was performed to examine logit-transformed consistency scores with and without an auditory cue (metronome vs. continuation conditions), with tempo (400, 550, 700) and group (rDCD, TD) as factors, and age as a covariate. Results revealed a significant effect of group, $F(1,40) = 5.25$, $p = .027$, $\eta^2 = .03$, with higher consistency in the TD group (rDCD = 0.58, SD = 0.09; TD = 0.64, SD = 0.08), and a significant effect of condition, $F(1,40) = 6.80$, $p = .013$, $\eta^2 = .03$, with higher consistency in metronome tapping (metronome = 0.82, SD = 0.09; continuation = 0.40, SD = 0.14). There was no significant effect of tempo, $F(1.97, 78.81) = 1.05$, $p = .352$, $\eta^2 = .006$ or interactions (condition x group: F(1,40) = 0.02, p = .89, $\eta^2 < .001$; tempo x group: $F(2, 80) = 0.32$, $p = .73$, $\eta^2 = .002$; condition x tempo: $F(2, 80) = 2.45$, $p = .09$, $\eta^2 = .013$; tempo x group x condition: $F(2, 80) = 0.56$, $p = .57$, $\eta^2 = .003$).

The Influence of Tempo on Tapping Production Accuracy

Phase accuracy scores during tapping production tasks were calculated using circular statistics to find the angle of the average vector *R* (Figure S4A). Scores were measured in radians. Tapping production was measured during three tempi (400, 550, and 700 ms inter-onset intervals). All sphericity and normality assumptions were met. To first compare just metronome and continuation with tempo as a factor, a 2x2x3 mixed design ANOVA with factors condition (metronome and continuation), tempo (400, 550, and 700 ms IOI), and group (rDCD and TD) with age as a covariate was performed. We found no main effects of condition, $F(1,40) = 0.24$, $p = .628$, $\eta^2 = .001$, group, $F(1,40) = 1.17$, $p = .285$, $\eta^2 = .006$, tempo, $F(2, 80) = 1.10$, $p = .338$, $\eta^2 = .007$, or interactions (Figure S4C).

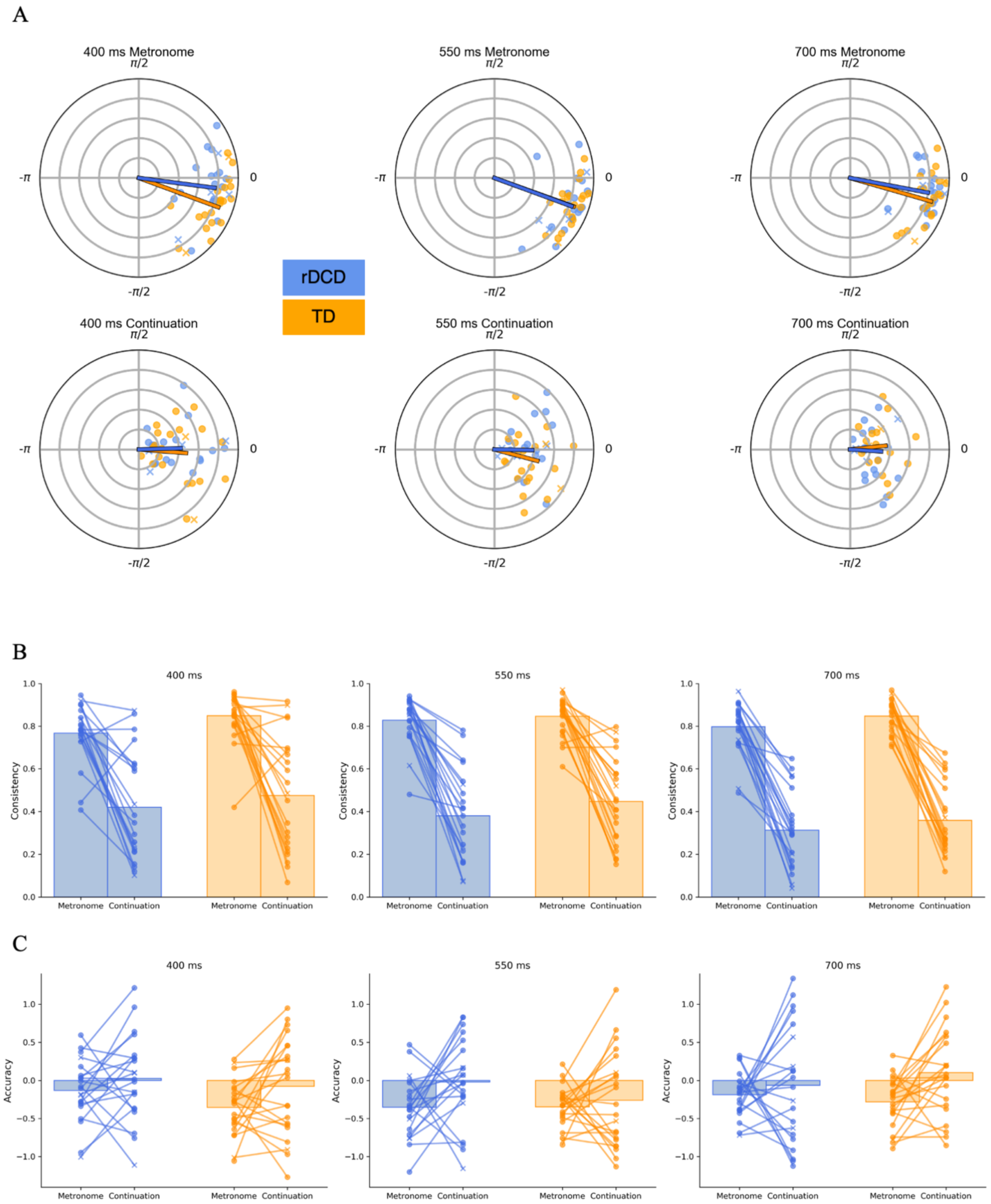

Figure S4. Consistency and phase accuracy scores for rDCD and TD groups in metronome and continuation tapping across three tempi. Participants with CPRS-R:S T-scores >60 are represented by Xs. (A) Polar plots of tapping production during metronome and continuation tapping, separated by tempo. Tapping consistency was calculated by the length of the mean vector R, and tapping accuracy was calculated by the angle of the mean vector R. (B) Tapping consistency separated by group and tempo. (C) Tapping accuracy (radians) separated by group and tempo. Negative values represent tapping before the beat.

The Influence of Song Excerpt on Consistency and Phase During Music Tapping

During music tapping production, participants tapped along to the beat of six different musical excerpts ranging in tempi from 500-700 ms IOIs. For consistency scores, a 2x6 mixed ANOVA with age as a covariate and factors group (rDCD and TD) and song (six excerpts) showed no significant effects of group ($F(1,35) = 1.08$, $p = .305$, $\eta^2 = .017$), song ($F(5, 175) = 1.05$, $p = .390$, $\eta^2 = .008$), or interactions. Consistency scores for both rDCD and TD groups, separated by song, are summarized in Figure S5A. For phase accuracy, a 2x6 mixed ANOVA with age as a covariate and factors group (rDCD and TD) and song (six excerpts) showed no effects of group ($F(1,36) = 0.008$, $p = .929$, $\eta^2 < .001$), song ($F(3.94, 141.7) = 0.34$, $p = .85$, $\eta^2 = .006$, or interaction. Greenhouse-Geisser corrections were applied to the phase data ($\varepsilon = .787$) as the assumption of sphericity was not met ($X^2 = 29.32$, $p = .010$). Accuracy scores are summarized in Figure S5B.

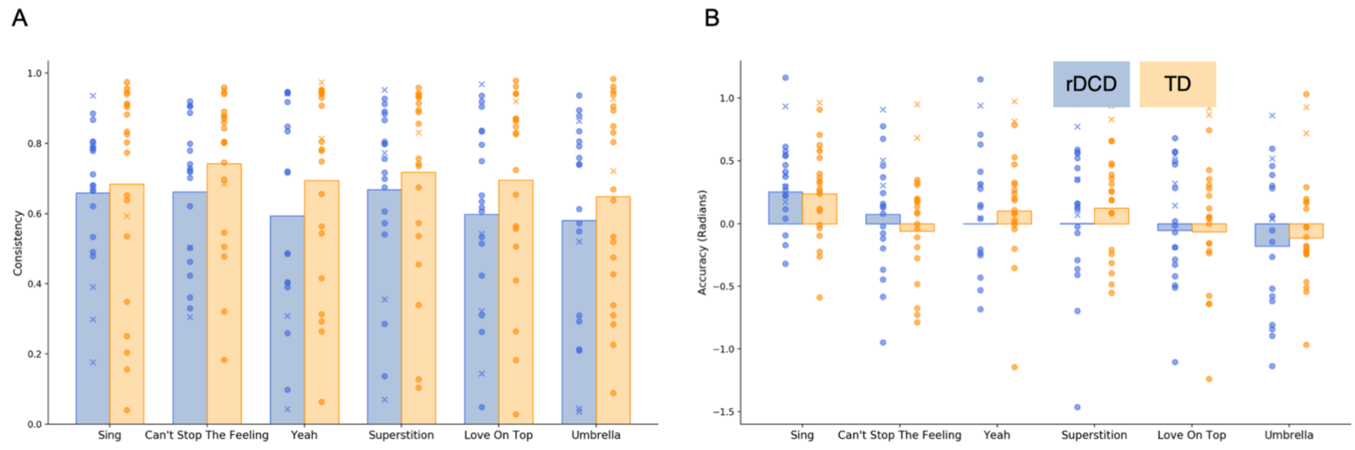

Figure S5. Consistency and Accuracy scores for rDCD and TD groups in music tapping for each of the six musical excerpts. Participants with CPRS-R:S T-scores >60 are represented by Xs. (A) Consistency scores during music tapping separated by song. (B) Phase accuracy scores (radians) during music tapping separated by song. Negative values represent tapping before the beat.

Results of Tapping Production Analyses if All Trials Kept

To account for trials where participants did not tap the beat of the song, but instead tapped a more complex rhythm, any trials flagged by the semi-professional musician rater as ‘rhythm tapping’ were removed. Given that these trials were flagged using the rater’s judgement, the analysis investigating tapping consistency across conditions was run with all trials included. An ANOVA with factors condition (metronome, music, and continuation) and group (rDCD, TD) and age as a covariate showed a significant effect of group, $F(1,40) = 5.67$, $p = .033$, $\eta^2 = .051$, a significant effect of condition, $F(1.52, 60.75) = 4.31$, $p = .027$, $\eta^2 = .040$, and no interaction, $F(1.52, 60.75) = 0.61$, $p = 0.505$, $\eta^2 = .006$. Greenhouse-Geisser corrections were applied ($\varepsilon = .759$) as the assumption of sphericity was not met ($X^2 = 14.86$, $p < .001$). T-tests between pairs of tapping conditions were significant, such that music tapping was significantly more consistent than continuation tapping ($t(42) = -4.75$, $p < .001$) and significantly less consistent than metronome tapping ($t(42) = 6.14$, $p < .001$). The correlation between MABC-2

scores and a general tapping score (all three conditions averaged for each participant) nearly reached significance, $r(40) = 0.34$, $p = .053$, after correcting for multiple comparisons.

Relations Between Each Set of Task Conditions

Pearson's correlation coefficients were calculated for each possible pair of tasks. For tapping production tasks, consistency scores were used to calculate correlations. For speech timing distortion tasks, proportion of correct responses were used. All correlation plots are shown in Figure S6. To further investigate the relations of these tasks with general motor skills, a tapping score (mean consistency of tapping production averaged across metronome, continuation, and music conditions) and scores on the modulated speech timing task were correlated with MABC-2 percentiles (Figure S7). To understand which of the tapping tasks were most related to the MABC-2 percentiles, each of the metronome, continuation, and music tapping tasks were correlated separately to the MABC-2, corrected for multiple comparisons (Bonferroni). Metronome tapping and MABC-2 percentiles were significantly correlated before Bonferroni correction ($r(41) = .34$, $p = .023$), but did not reach significance after correction ($p_{corr} = .085$). Continuation tapping and MABC-2 percentiles were not significantly correlated ($r(41) = .25$, $p_{corr} = .324$), nor were music tapping and MABC-2 percentiles ($r(41) = .22$, $p_{corr} = .810$).

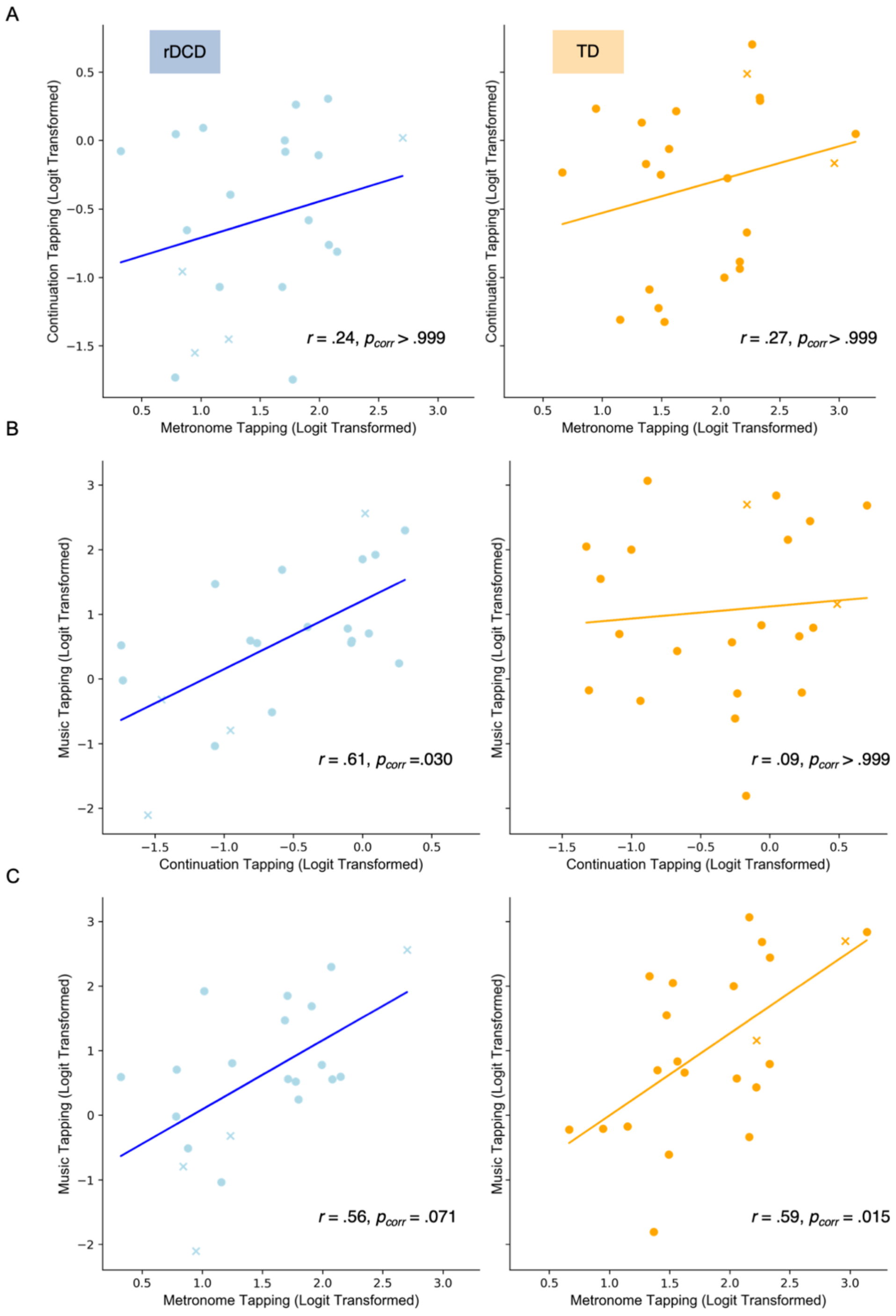
A
rDCD
TD
Continuation Tapping (Logit Transformed)
Metronome Tapping (Logit Transformed)
r = .24, pcorr > .999
r = .27, pcorr > .999
B
Music Tapping (Logit Transformed)
Continuation Tapping (Logit Transformed)
r = .61, pcorr =.030
r = .09, pcorr > .999
C
Music Tapping (Logit Transformed)
Metronome Tapping (Logit Transformed)
r = .56, pcorr = .071
r = .59, pcorr = .015

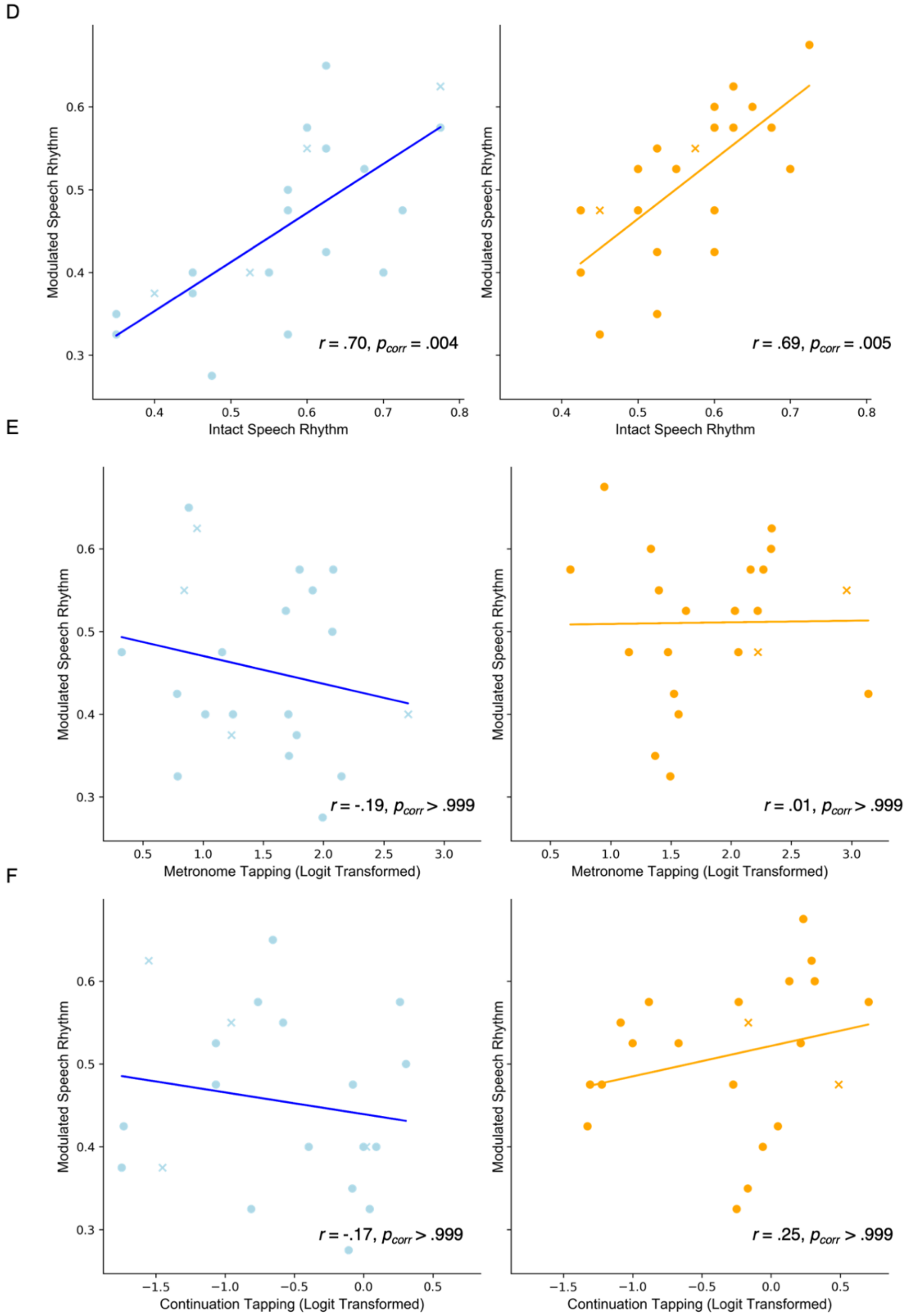
D
Modulated Speech Rhythm
Intact Speech Rhythm
r = .70, p_corr = .004
r = .69, p_corr = .005
E
Modulated Speech Rhythm
Metronome Tapping (Logit Transformed)
r = -.19, p_corr > .999
r = .01, p_corr > .999
F
Modulated Speech Rhythm
Continuation Tapping (Logit Transformed)
r = -.17, p_corr > .999
r = .25, p_corr > .999

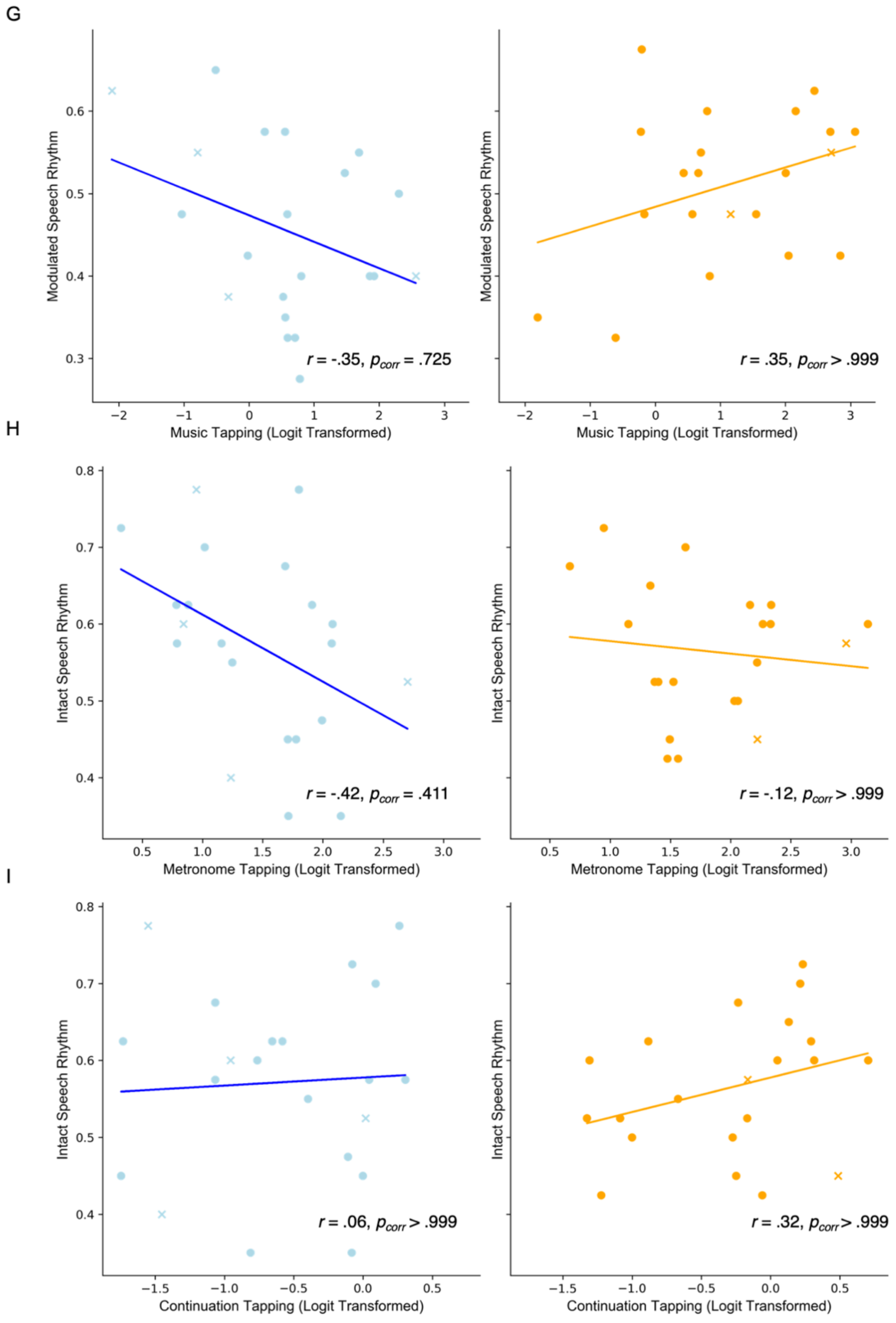
G
Modulated Speech Rhythm
Music Tapping (Logit Transformed)
r = -.35, p_corr = .725
r = .35, p_corr > .999
H
Intact Speech Rhythm
Metronome Tapping (Logit Transformed)
r = -.42, p_corr = .411
r = -.12, p_corr > .999
I
Intact Speech Rhythm
Continuation Tapping (Logit Transformed)
r = .06, p_corr > .999
r = .32, p_corr > .999

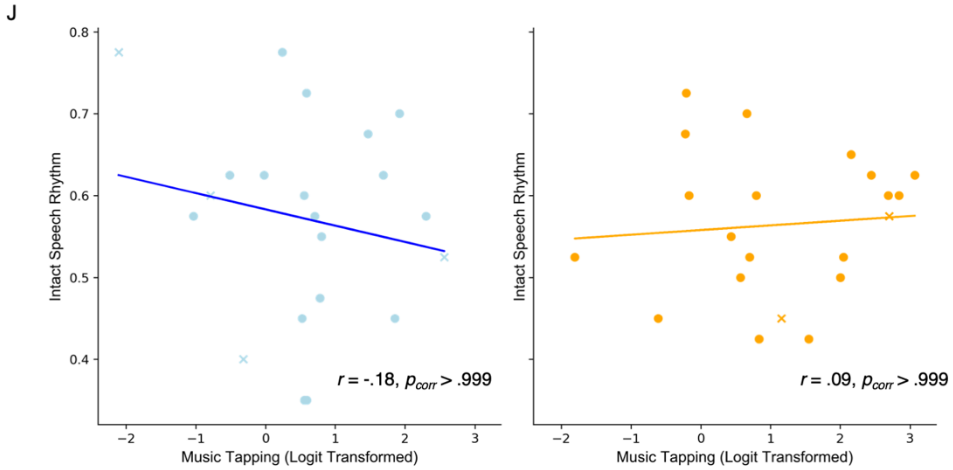

Figure S6. Correlation plots of each pair of tapping production conditions (metronome, music, and continuation) and speech recognition with distorted timing (intact rhythm, modulated rhythm). All correlations are separated by group (rDCD in blue), and the plotted line represents the line of best fit. Participants with CPRS-R:S T-scores >60 are represented by Xs. (A) Metronome and Continuation Tapping. (B) Continuation and Music Tapping. (C) Metronome and Music Tapping. (D) Intact Speech and Modulated Speech. (E) Metronome Tapping and Modulated Speech. (F) Continuation Tapping and Modulated Speech. (G) Music Tapping and Modulated Speech. (H) Metronome Tapping and Intact Speech. (I) Continuation Tapping and Intact Speech. (J) Music Tapping and Intact Speech.

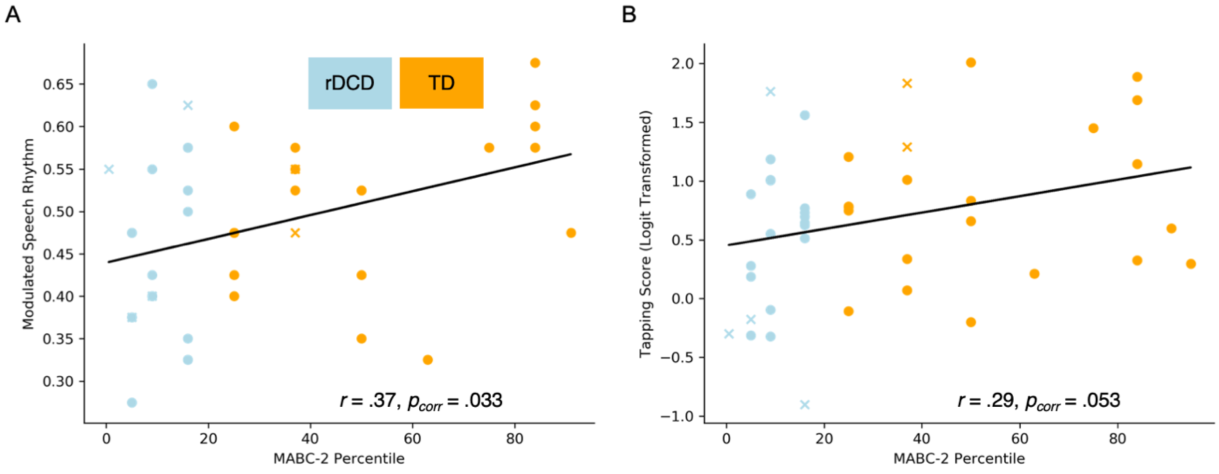

Figure S7. Correlation plots of MABC-2 scores with modulated speech rhythm scores and tapping scores. Tapping score is calculated as the average consistency in music, continuation, and metronome tapping. Groups are combined; rDCD is represented in blue, TD is represented in orange. Participants with CPRS-R:S T-scores >60 are represented by Xs. (A) MABC-2 percentile and proportion of correct responses in the modulated speech rhythm condition. (B) MABC-2 percentile and tapping score.

Relation of Semi-Professional Musician Ratings to Circular Statistics

To ensure quality of the circular statistical analyses performed on the consistency data, a semi-professional musician rated every music tapping trial on a scale of 0-100 for the question: How synchronized was the tapping to the music, regardless of whether they tapped the beat or the rhythm? Results showed a significant positive correlation between the ratings of synchronization and the calculated consistency scores, $r(512)=0.75$, $p < .001$.

Influence of Music Training on Task Performance

To ensure that the group effects present in the speech recognition with distorted timing were not affected by music training, the ANOVA was run including years of music training as a covariate. Results showed a trend towards an effect of music training ($F(1,38) = 3.89$, $p = .056$, $\eta^2 = .071$), but the interaction of group and condition remained significant ($F(1,38) = 6.12$, $p = .018$, $\eta^2 = .023$), and the main effects of group ($F(1,38) = 0.91$, $p = .346$, $\eta^2 = .017$) and condition ($F(1,38) = 0.02$, $p = .884$, $\eta^2 < .001$) remained insignificant. To ensure the group effects present in the tapping tasks were not affected by music training, the ANOVA was run including years of music training as a covariate. The effect of music training was not significant ($F(1,39) = 0.04$, $p = .840$, $\eta^2 < .001$) and the effect of group remained significant ($F(1,39) = 4.36$, $p = .043$, $\eta^2 = .046$), as did the effect of condition ($F(1.63, 63.37) = 5.10$, $p = .013$, $\eta^2 = .047$).

References

1. Cairney, J. *et al.* The Coordination and Activity Tracking in CHildren (CATCH) study: rationale and design. *BMC Public Health* **15**, 1266 (2015).

2. Cairney, J. *et al.* Cohort profile: the Canadian coordination and activity tracking in children (CATCH) longitudinal cohort. *BMJ Open* **9**, e029784 (2019).

3. Henderson, S, Sugden, D., & Barnett, A. Movement Assessment Battery for Children Examiner's Manuel. 2nd ed. *London: Harcourt Assessment* (2007).
